# S100A8/S100A9 Integrates F-Actin and Microtubule Dynamics to Prevent Uncontrolled Extravasation of Leukocytes

**DOI:** 10.3390/biomedicines11030835

**Published:** 2023-03-09

**Authors:** Marc Wolf, Robiya Joseph, Judith Austermann, Chiara Scharrnbeck-Davis, Sven Hermann, Johannes Roth, Thomas Vogl

**Affiliations:** 1 Institute of Immunology, University of Münster, 48149 Münster, Germany; marcwolf@uni-muenster.de (M.W.); judith.austermann@ukmuenster.de (J.A.); rothj@uni-muenster.de (J.R.); 2 Department of Gynecologic Oncology and Reproductive Medicine, The University of Texas, Houston, TX 77030, USA; rjoseph7@mdanderson.org; 3 European Institute for Molecular Imaging, University of Münster, 48149 Münster, Germany; shermann@uni-muenster.de

**Keywords:** calprotectin, cytoskeleton, integrin, leukocyte, migration

## Abstract

Immune reactions are characterized by the rapid immigration of phagocytes into sites of inflammation. Meticulous regulation of these migratory processes is crucial for preventing uncontrolled and harmful phagocyte extravasation. S100A8/S100A9 is the major calcium-binding protein complex expressed in phagocytes. After release, this complex acts as a proinflammatory alarmin in the extracellular space, but the intracellular functions of these highly abundant proteins are less clear. Results of this study reveal an important role of S100A8/S100A9 in coordinated cytoskeleton rearrangement during migration. We found that S100A8/S100A9 was able to cross-link F-actin and microtubules in a calcium- and phosphorylation-dependent manner. Cells deficient in S100A8/S100A9 showed abnormalities in cell adhesion and motility. Missing cytoskeletal interactions of S100A8/S100A9 caused differences in the surface expression and activation of β1-integrins as well as in the regulation of Src/Syk kinase family members. Loss of S100A8/S100A9 led to dysregulated integrin-mediated adhesion and migration, resulting in an overall higher dynamic activity of non-activated S100A8/S100A9-deficient phagocytes. Our data suggest that intracellular S100A8/S100A9 is part of a novel regulatory mechanism that ensures the precise control necessary to facilitate the change between the quiescent and activated state of phagocytes.

## 1. Introduction

Directional cell migration of phagocytes during inflammation allows for their rapid accumulation at sites of injury and infection. Neutrophils and monocytes can migrate exceptionally fast and are the first immigrating cells after the onset of inflammation. In order to transmigrate, these cells have to permanently remodel their cytoskeleton. This process involves the orchestrated interplay of intracellular signaling pathways, which leads to the activation of specific protein kinases and the transient elevation of intracellular calcium concentrations [1,2,3,4].

The important role of the actin-based cytoskeleton and its regulation during adhesion and migration is well-characterized [5,6], but much less is known about the involvement of the other two major cytoskeletal components (intermediate filaments and microtubules (MTs)) and their interplay with the actin-based cytoskeleton. Phagocytes are characterized by a high turnover of MTs during transmigration [1,7], and reorganization of MTs is controlled by the phosphorylation of specific MT-associated proteins and by the modulation of intracellular calcium levels [8,9]. The elevation of intracellular calcium concentrations usually induces conformational changes in the calcium-binding proteins, which in turn allows them to interact with distinct intracellular targets.

The major calcium-binding molecules expressed in neutrophils and monocytes are two members of the S100 protein family: S100A8 (also known as MRP8) and S100A9 (also known as MRP14) [10]. The heterodimer S100A8/S100A9 is the predominant intracellular form, and it constitutes up to 40% of neutrophils and 4–5% of monocyte cytosolic proteins [11]. Increased local and systemic levels of released S100A8/S100A9 occur in many inflammatory disorders, and it is well-accepted that secreted S100A8/S100A9 exerts proinflammatory effects and contributes to disease progression [10,12,13,14,15].

To date, most studies have focused on the extracellular role of S100A8/S100A9, and much less is known about the intracellular function(s) of this protein complex. It was recently reported that S100A9 was associated with cortical F-actin in human neutrophils, and calcium-induced tetramers of S100A8/S100A9 were shown to induce tubulin polymerization, bind MTs in vitro, and co-localize with MTs during the activation of monocytes [16,17,18,19,20]. Furthermore, it was reported that phosphorylation of S100A9 at Thr113 by the p38 mitogen-activated protein kinase (MAPK) played a pivotal role in modulating this interaction [16]. However, the functional consequences of S100-MT/S100-actin interactions and their impact on cytoskeletal properties, transmigration, and adhesion are still unclear.

Migratory processes require tightly controlled and regulated dynamic interactions between the cell and its surrounding substrate, which are usually mediated by specialized adhesion receptors such as selectins and integrins. These adhesion receptors link the cell to the extracellular matrix and transmit signals necessary for proper cell polarization and finally locomotion [21,22]. Although much is already known about the “leukocyte adhesion and transmigration cascade” that enables extravasation and even reverse migration [23,24], the role of the cytoskeleton in maintaining the resting state of phagocytes to prevent uncontrolled and detrimental extravasation is still unknown.

In this study, we assessed how intracellular S100A8/S100A9 influences the stability and repolymerization of MTs in living cells. We analyzed the function of S100A8/S100A9 complexes and investigated how S100A8/S100A9 deficiency affects cell polarization, adhesion, and the migration of phagocytes.

## 2. Materials and Methods

### 2.1. Cells, Transfection, Stimulation, and Mice

For the migration and adhesion assays, we used freshly isolated bone marrow cells (BMCs) from the femur and tibia of wildtype and S100A9 -/- mice (C57BL/6 strain). Mice were bred and housed under specific pathogen-free conditions and used at the age of 8–12 weeks. S100A9 -/- mice were generated by targeted gene disruption, as described previously [25]. Expression analyses revealed that these mice are also deficient in S100A8 at the protein level, although the S100A8-mRNA levels are not altered. Therefore, these mice and cells are considered to be functional S100A8 and S100A9 double knockouts. The isolation of BMCs was carried out as described elsewhere and were cultured in Dulbecco’s MEM with 20% L-cell SN, 2% L-glutamine, 1% Pen/Strep, and 10% FCS (*v*/*v*, heat inactivated) [25]. We subsequently characterized isolated bone marrow cells by fluorescence activated cell sorting (FACS) analyses using anti-Gr-1 (BD Biosciences, Heidelberg, Germany), anti-Ly6C (BioLegend, Fell, Germany), and anti-Ly6G (eBioscience, Frankfurt, Germany) antibodies. About 65% of the whole BMC population was Gr-1 positive. No differences in Ly6C (69 ± 3%) and Ly6G (76 ± 2%) surface expression between S100A9 -/- and wildtype BMC were detected. For the transendothelial migration experiments, we used murine bEND5 cells to provide a confluent endothelial cell layer.

To analyze the intracellular function and impact of S100A8/S100A9 on the cytoskeletal dynamics, we stably transfected HEK293 and NIH 3T3 cells with a pVITRO2mcs (InvivoGen, San Diego, CA, USA) mammalian expression vector construct containing the coding sequences of human S100A8 (5′AgeI; 3′BamHI) and S100A9 (5′Xho; 3′Nhe1). HEK293 and NIH 3T3 were chosen for these type of experiments because they are large and flat, and therefore it is much easier to display submolecular structures compared to much smaller primary bone marrow cells. Both cell lines were cultured in Dulbecco’s MEM with 2% L-glutamine, 1% Pen/Strep, and 10% FCS (*v*/*v*, heat inactivated) in 20 cm^2^ culture flasks at 37 °C. A total of 50 μg/mL hygromycin was strictly used for all cultures. Transfections were carried out using the PolyFect^®^ (Qiagen, Hilden, Germany) transfection reagent according to the manufacturer’s instructions. Untransfected or empty vector mock-transfected cells served as the controls and hygromycin was used for the selection of positive clones.

In some experiments, we stimulated cells for 4 h with 10 nM phorbol 12-myristate 13-acetate (PMA) (Sigma-Aldrich, Munich, Germany) to induce cell activation and membrane ruffling.

### 2.2. Immunofluorescence Microscopy

Cells were cultured overnight on fibronectin-coated Lab-Tek™ chamber slides (Nunc, Wiesbaden, Germany) and fixed in 4% paraformaldehyde for 30 min at room temperature. After cell permeabilization with 0.1% Triton X-100 in phosphate-buffered saline (PBS) for 25 min at room temperature, the unspecific binding of antibodies was blocked with 3% bovine serum albumin (BSA) in PBS for 2 h at room temperature prior to adding primary antibodies at 4 °C overnight. We used the following antibodies for the different stainings: mouse anti-α-tubulin [DM-1A] (MP Biomedicals, Illkirch, France), rabbit anti-actin22-33 (Sigma-Aldrich), rabbit anti-paxillin [Y113] (Abcam, Cambridge, UK), mouse anti-β1-integrin [HUTS-4] for the detection of activated β1-integrin (Millipore, Schwalbach Germany), rabbit anti-total-β1-integrin (a kind gift from J. Eble, University of Münster, Germany), and rabbit anti-S100A9 (generated in our institute). F-actin was visualized via staining with fluorescein isothiocyanate (FITC)-conjugated phalloidin (Sigma-Aldrich).

Primary antibody incubation was followed by 2 h incubation at room temperature with corresponding Cy3-, FITC-, TRITC-, or DyLight649-conjugated secondary antibodies (all from Dianova, Hamburg, Germany), as indicated in the figures. Subsequently, slides were washed thoroughly in PBS and mounted with Dako^®^ Fluorescent Mounting Medium (Dako, Hamburg, Germany).

We analyzed specimens under a Zeiss AxioObserver Z1 inverted microscope using an EC Plan-Neofluar 40×/1.3 or a Plan-Apochromat 63×/1.4 oil immersion objective (Carl Zeiss MicroImaging, Göttingen, Germany). Optical sections were acquired using the Zeiss ApoTome structured illumination system. Images were recorded with an AxioCAM MRm and processed with Zeiss AxioVision 4.8.1.

### 2.3. CS-T/CS Fixation

S100A8/S100A9-expressing HEK293 cells were cultured as described above and stimulated for 4 h with 10 nM PMA prior to fixation. Cells were washed with PBS, permeabilized for 1 min in CS buffer (10 mM Hepes, pH 6.8, 100 mM KCl, 3 mM MgCl_2_, 200 mM saccharose, and 1 mM phenylmethylsulfonyl fluoride) containing 0.5% Triton X-100 (to make CS-T buffer) and 20 µM taxol for microtubule stabilization, and washed twice in CS buffer for 5 min each time. After fixation in 4% formaldehyde in PBS for 4 min and methanol for 6 min at −20 °C, we processed the slides for double-labeling immunofluorescence, as described above using antibodies against S100A9 and α-tubulin.

### 2.4. MT de-/Repolymerization Assay

We treated S100A8/S100A9 and mock-transfected HEK293 cells with 10 µM nocodazole (Sigma-Aldrich) in the cell culture medium for 0, 10, 20, and 30 min, respectively, to assess the MT stability. After the incubation periods, cells were immediately fixed with 4% paraformaldehyde and stained for α-tubulin, as described above. To analyze the influence of S100A8/S100A9 on MT reformation, cells were pretreated with 10 µM of nocodazole in cell culture medium for 1 h to completely depolymerize MTs. Repolymerization was induced by nocodazole wash out with fresh nocodazole-free cell culture medium, and documented via α-tubulin antibody staining (see above) after 0, 5, 10, and 15 min of repolymerization.

### 2.5. MT/F-Actin Spin-Down Binding Assay

MTs and actin filaments were preassembled according to the manufacturer’s instructions for the Fluorescent Microtubule Biochem Kit and Actin Binding Protein Biochem Kit (both from Cytoskeleton, Denver, CO, USA), respectively. We investigated binding to F-actin by adding S100A8/S100A9 or S100A8/phospho-A9 complexes in the absence (addition of 1 mM EGTA) or presence of 100 µM calcium. After incubation for 30 min at room temperature, samples were centrifuged at 100,000× *g* for 1 h and further processed for analysis (see below).

We assessed the cross-linking activity by mixing equal volumes of MTs and F-actin followed by the addition S100A8/S100A9 or S100A8/phospho-S100A9 complexes in the absence (addition of EGTA) or presence of 100 µM calcium. Samples were loaded carefully onto a cushion buffer (5 mM Tris pH8, 30% glycerol) after incubation for 30 min at room temperature and centrifuged at 10,000× *g* for 1 h. We carefully adjusted the composition of the cushion buffer and the rotational speed to spin-down MTs and attached molecules while keeping non-bound smaller proteins and actin filaments in the supernatant fraction. Pellets and supernatants of both assay types (actin binding and cross-linking activity) were analyzed by SDS-PAGE, followed by Coomassie staining. Bands were quantified using the software Lumi-Analyst 3.0 of the Lumi-Imager F1 (Boehringer, Mannheim, Germany).

### 2.6. MT/Actin Fluorescence In Vitro Assay

Fluorescently labeled MTs were preassembled for 30 min at 37 °C under tubulin-polymerizing conditions (tubulin polymerization buffer (G-PEM): 80 mM PIPES, 2 mM MgCl_2_, 1.6 M glycerol, 1 mM GTP, pH 6.9). Subsequently, the formed microtubules were stabilized with 20 µM taxol according to the manufacturer’s instructions for the Fluorescent Microtubule Biochem Kit (Cytoskeleton) with the modification of using EGTA-free buffers. F-actin was assembled according to the manufacturer’s instructions for the Actin Binding Protein Biochem Kit (Cytoskeleton). Polymerized F-actin was subsequently stabilized and fluorescently labeled by adding 1 µM FITC-phalloidin (Sigma-Aldrich). We investigated binding to and cross-linking of MTs and F-actin using mixtures of both filaments after the addition of S100A8/S100A9 or S100A8/phospho-A9 complexes in the absence (addition of EGTA) or presence of 100 µM calcium. BSA served as the negative control, and α-actinin served as the positive control. Samples were incubated for 30 min at room temperature, diluted, and immediately analyzed by fluorescence microscopy.

### 2.7. Duolink^®^ Assay

To identify protein–protein interactions, we conducted proximity ligation assays (PLA, DuoLink II^®^; Olink Bioscience, Uppsala, Sweden) according to the manufacturer’s instructions. Briefly, cells were cultured, treated, and fixed as described above or in the figures. To detect possible protein–protein interactions, we used the following antibody combinations in the PLA: mouse anti-α-tubulin/rabbit anti-S100A9, mouse anti-β-actin/rabbit anti-S100A9, and mouse anti-α-tubulin/rabbit anti-actin22-33. The antibody combination of mouse anti-human S100A8/rabbit anti-human S100A9 served as the negative control for mock-transfected NIH 3T3 cells and as the positive control for S100A8/S100A9-transfected NIH 3T3 cells. The PLA probes anti-mouse PLUS and anti-rabbit MINUS were used as secondary antibodies. To quantify unspecific background signals, we omitted primary antibodies in some experiments. Hybridization of the two PLA probes only occurs when the two proteins of interest are in close proximity (<40 nm). Hybridization yields a detectable spot-like signal after a subsequent amplification step, for which the Duolink^®^ Detection Kit 563 was used. Specimens were mounted with Dako^®^ Fluorescent Mounting Medium and analyzed under a Zeiss AxioObserver Z1 inverted microscope. Each visible spot represents protein–protein interactions.

### 2.8. Flow Cytometry

The cells (0.5 × 10^6^ cells in 100 μL media) were centrifuged down at 1100× *g* rpm for 1 min at 4 °C. The supernatant was discarded and the cells were blocked with 1% BSA (in PBS) for 30 min on ice. The cells were centrifuged again at 1100× *g* rpm for 1 min at 4 °C. The supernatant was removed and the appropriate antibodies were added in 1:50 dilution in PBS and incubated on ice for 30 min. To quantify the surface expression of various integrins and selectins, we incubated HEK293 cells (S100A8/S100A9 as well as mock-transfected cells) or freshly isolated murine BMCs from the wildtype and S100A9 -/- mice with antibodies against CD11a (LFA-1), CD11b (Mac1), CD11c (αX), CD18 (β2), CD29 (β1), human or murine CD49d (VLA-4α) (ImmunoTools, Friesoythe, Germany), CD54 (ICAM-1), and CD106 (VCAM-1), respectively, followed by incubation with the corresponding FITC-, PE-, or APC-conjugated secondary antibodies (Dianova, Hamburg, Germany). Isotype matched polyclonal antibodies (Sigma-Aldrich) served as the controls. Cells were analyzed using a FACSCalibur flow cytometer (BD Biosciences), and data were evaluated using CellQuestPro and WinMDI software.

### 2.9. Adhesion Assay

Freshly isolated BMCs from wildtype and S100A9 -/- mice were seeded onto either uncoated or VCAM1-coated (1 µg/mL, 16 h, 37 °C) 24 well-plates (4 × 10^5^ cells per well) and allowed to settle for 1 h. In additional experiments, we used S100A8/S100A9- or the mock-transfected HEK293 and NIH 3T3 cell lines. Cells were washed twice with PBS at 37 °C to remove non-adherent cells and fixed with 2% fresh glutaraldehyde for 10 min at room temperature. The fixed cells were stained with 0.5% crystal violet in 200 mM boric acid (pH 8.0) for 15 min at room temperature and then washed 3–4 times with deionized water. Cells were lysed with 10% acetic acid, and the optical density was measured at 560 nm using a Dynatech MRX microplate reader (Dynex Laboratories, Denkendorf, Germany). In additional experiments, adherent BMCs were resuspended and subsequently characterized by FACS analysis using anti-Gr-1, anti-Ly6C, and anti-Ly6G antibodies. Equal fractions of Ly6C- and Ly6G-positive adherent cells were observed by flow cytometry.

### 2.10. Src/Syk Expression and Western Blotting

Preparation of the whole cell extracts for Western blot analysis was performed as described earlier [16]. Equal amounts of proteins (10 µg) per lane were separated on 12% polyacrylamide gels, transferred onto nitrocellulose membranes (Thermo Scientific, Waltham, MA, USA), and processed for antibody staining. We labeled membranes with antibodies against the total (Src Rabbit mAb) and phospho-Src (P-Src Family (Tyr416) Rabbit mAb) or total (Syk Rabbit Ab) and phospho-Syk (Tyr519/520, P-Syk Rabbit Ab), respectively, and the labeling of total p38 (p38 MAPK Rabbit Ab) served as an additional loading control (all from Cell Signaling Technology, Danvers, MA, USA). Finally, we stained the membranes with corresponding horseradish peroxidase-coupled secondary antibodies (Dianova, Hamburg, Germany), visualized them via enhanced chemiluminescence (Amersham Pharmacia Biotech), and documented the results using the Bio-Rad ChemiDoc™XRS+ system (Bio-Rad Laboratories, Munich, Germany).

### 2.11. Real-Time Reverse Transcription Polymerase Chain Reaction (RT-PCR)

For the RT-PCR analyses, we analyzed RNA isolated from the wildtype and S100A9 -/- BMCs in duplicate. cDNA was synthesized from 1 µg of total RNA using SuperScriptII RNase-H reverse transcriptase (Invitrogen, Waltham, MA, USA). The primers used for PCR analysis were as follows: Hck (Src Family Kinase; forward primer: GAC TTT GAC CCC CAG CAC GGA GAC; reverse primer: CAC ACT TCT CCA AAC TGC CCA) and Syk (forward primer: TCC ATG GCA ACA TCT CCA G; reverse primer: GAC ATG GTA CCG TGA GGA). RT-PCR was performed using the QuantiTect SYBR Green PCR Kit (Qiagen) according to the manufacturer’s instructions. Samples were subjected to initial denaturation at 95 °C for 15 min and 40 cycles of PCR (94 °C for 15 s, 60 °C for 30 s, and 72 °C for 30 s). We used GAPDH as the housekeeping gene (forward primer: CCA CCC CAG CAA GGA CAC T; reverse primer: TCC CTA GGC CCC TCC TGT TAT).

### 2.12. Transmigration Assay

We performed two chamber transmigration assays in the absence and presence of murine endothelial cells (bEND5) that were grown to confluency (~2–3 days) on 5 µm pore size Transwell filters (Costar, Bodenstein, Germany) as described elsewhere [26]. Neither filters were coated by any adhesion molecules nor the endothelial monolayer pre-activated by any chemokines/cytokines before adding the cells. Freshly harvested BMCs from wildtype and S100A9 -/- mice were added to the upper chamber (1 × 10^6^ cells/100 µL BMC medium) and allowed to transmigrate for 4 h at 37 °C. After incubation, the whole experimental plate was kept on ice for 20 min. Later, the chamber was rinsed with 200 μL of ice-cold PBS and the number of migrated cells counted using a CASY cell counter (Schärfe System, Reutlingen, Germany). Experiments were performed in quadruplicate. We verified the integrity of the bEND5-endothelial monolayer morphologically and by measuring transendothelial resistance using an EVOM^TM^ epithelial volt-ohmmeter (World Precision Instruments, Sarasota, FL, USA) before starting the experiment.

### 2.13. Blocking Experiments

To analyze the involvement of CD49d in adhesion and transmigration, freshly isolated BMCs from the wildtype and S100A9 -/- mice were preincubated for 30 min with 10 µg/mL of the CD49d blocking antibodies R1-2 (BD Biosciences) or natalizumab (a kind gift from L. Klotz, University of Münster, Germany) and used for the adhesion or transmigration studies as described above. Likewise, cells pretreated for 30 min with 10 µM of the Src inhibitor 4-amino-5-(4-methylphenyl)-7-(t-butyl)pyrazolo-d-3,4-pyrimidine (PP1); Calbiochem, Darmstadt, Germany), 2 µM of the Syk inhibitor 2-(2-Aminoethylamino)-4-(3-trifluoromethylanilino)-pyrimidine-5-carboxamide (DHC), Calbiochem), or a combination of both were used to block Src and Syk kinase activation.

### 2.14. Two-Dimensional (2D) Migration Assay

We conducted 2D under agarose migration assays as described elsewhere [27]. Briefly, 35 mm glass bottom Petri dishes (ibidi, Martinsried, Germany) were coated with 50 µg/mL of fibronectin (BD) for 1 h at 37 °C, washed twice with sterile water, and dried prior to casting the agarose gel. Next, 2% (*w*/*v*) agarose was boiled in plain RPMI1640 medium (Biochrom, Berlin, Germany) without any supplements and mixed with an equal amount of preheated (50 °C) complete RPMI1640 medium to yield a final agarose concentration of 1% (*w*/*v*). Finally, 2 mL of the warm and still fluid mixture were quickly added to the culture dish, covered with the dish lid, and allowed to solidify at room temperature.

To assess migration, two wells were punched 1.5 mm apart using a template and a 3 mm sterile skin punch. The prepared culture dishes were then equilibrated for 1 h at 37 °C and 5% CO_2_ prior to seeding of the cells. Freshly isolated BMCs (~2000 cells) were seeded into the first well and allowed to settle for 20 min. Subsequently, 10 µg/mL of the chemoattractant LTB_4_ (Cayman Chemical, Ann Arbor, MI, USA) was added to the second well, and the dish was carefully placed onto a heated microscope stage (35 °C) with additional CO_2_ gassing (5%). Movement of cells was recorded at 1 frame per minute with a Zeiss AxioCAM MRm for a total period of 90 min.

### 2.15. Statistical Analysis

Statistically significant differences between treatments were identified using the Mann–Whitney U test for values with nonparametric distribution or the Student’s *t*-test for normally distributed values. *p* < 0.05 was considered to be statistically significant. All results shown are the mean ± standard deviation (SD).

## 3. Results

### 3.1. S100A8/S100A9 Affected the Dynamic Rearrangement of MTs in S100A8/S100A9-Expressing HEK293 Cells

**Figure 1:**
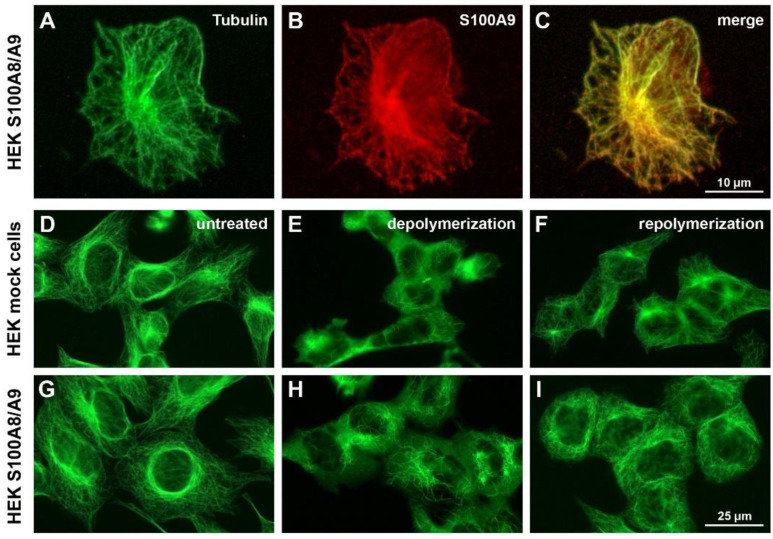
S100A8/S100A9 co-localized with MTs in S100A8/S100A9-transfected HEK293 cells. Double labeling immunofluorescence studies using anti-tubulin (**A**) and anti-S100A9 (**B**) antibodies showed clear co-localization (**C**) of S100A9 with MTs in PMA-stimulated (4 h/10 nM) cells after CS-T/CS fixation. Additionally, S100A8/S100A9 stabilized MTs and promoted their repolymerization (**D**–**I**). S100A8/S100A9-transfected HEK293 cells (**H**) showed more intact MTs after nocodazole treatment for 10 min in comparison to the mock-transfected control cells (**E**) as well as increased MT repolymerization 15 min after nocodazole washout (**I**). While the mock-transfected control cells (**F**) exhibited a MT cytoskeleton after repolymerization similar to that of the untreated cells (**D**), the transgenic cells (**I**) were characterized by a transient high amount of densely packed MTs compared to the corresponding untreated cells (**G**). Three independent experiments were performed and 25 cells per experiment were analyzed for each individual condition.

We first analyzed the expression pattern and subcellular localization of S100A8/S100A9 in stably transfected HEK293 cells via immunofluorescence microscopy. As expected, clear co-localization of S100A9 and MTs was observed after PMA activation (Figure 1A–C), which confirmed previous findings in human monocytes [19]. Anti-S100A8 antibody staining revealed an identical expression pattern.

We next investigated the possible consequences of this interaction with respect to MT stability and MT polymerization. S100A8/S100A9-expressing as well as mock-transfected control cells were treated with nocodazole, and the breakdown of the MT cytoskeleton was analyzed over time. The overall MT morphology of untreated S100A8/S100A9-expressing HEK cells was not altered compared to the control cells (Figure 1D,G), and both cell lines showed increasing MT depolymerization over time during nocodazole treatment. However, S100A8/S100A9-transfected cells were more resistant to nocodazole treatment, as indicated by more intact and longer MTs after 10 min of depolymerization compared to the mock-transfected cells (Figure 1E,H). After 30 min, the MT cytoskeleton of both cell lines was completely depolymerized.

Subsequently, we investigated the repolymerization of MTs after complete nocodazole-induced MT depolymerization followed by nocodazole washout. Mock-transfected control cells exhibited a perfectly rebuilt MT cytoskeleton after 15 min, which resembled that of the untreated cells (Figure 1F). In contrast, the S100A8/S100A9-expressing cells showed a more rapid MT repolymerization rate that was already visible after 5 min of repolymerization and most pronounced after 15 min (Figure 1I). However, the rebuilt MT cytoskeleton in the S100A8/S100A9-transfected cells was rough and disordered and characterized by a high amount of densely packed MTs. These data are in accordance with previous biochemical studies demonstrating that S100A8/S100A9 efficiently promotes tubulin polymerization in vitro [16].

### 3.2. S100A8/S100A9 Bridged Actin Filaments and MTs in a Calcium- and Phosphorylation-Dependent Manner

**Figure 2:**
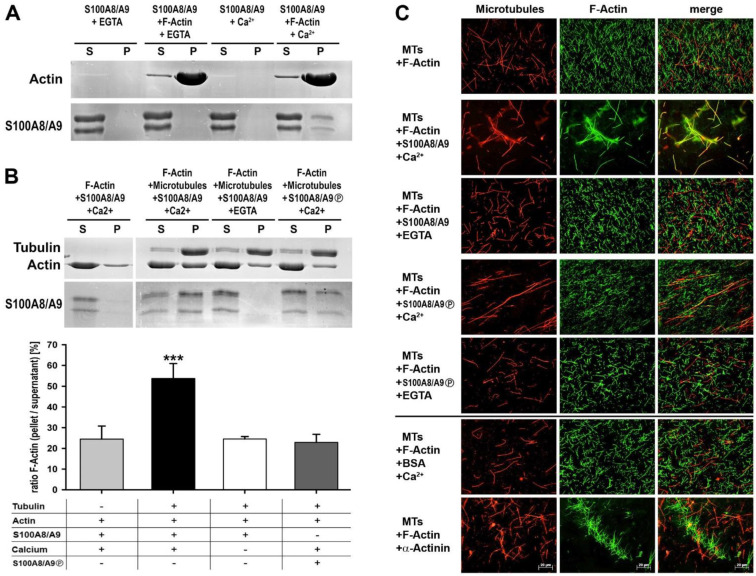
S100A8/S100A9 cross-linked actin filaments and MTs. (**A**) Co-sedimentation assay of prepolymerized F-actin in the presence of S100A8/S100A9 analyzed by SDS-PAGE (S = supernatant; P = pellet). S100A8/S100A9 in its calcium-loaded state bound to F-actin and was subsequently found in the pellet fraction after centrifugation (100,000× *g*/1 h). (**B**) Co-sedimentation assay and densitometric analysis of prepolymerized MTs in the presence of S100A8/S100A9 or S100A8/phospho-S100A9 and actin filaments analyzed by SDS-PAGE (centrifugation conditions 10,000× *g*/1 h). Only calcium-loaded S100A8/S100A9 caused increased sedimentation of F-actin, indicating MT/F-actin cross-linking. On the other hand, MT/F-actin cross-linking was diminished by the phosphorylation of the S100A8/S100A9 complex. (**C**) The fluorescence in vitro assay confirmed cross-linking of rhodamine-labeled MTs (red) and FITC-phalloidin labeled F-actin (green) by the S100A8/S100A9 complexes. It also revealed parallel alignment of the individual filaments. Again, only calcium-loaded S100A8/S100A9 was able to cross-link F-actin and MTs, while the phosphorylation of S100A9 abrogated cross-linking under the same conditions. BSA served as the unspecific negative control and α-actinin, which is known to bundle F-actin but not MTs, served as the positive control. (*n* = 3; mean ± SD; *** *p* < 0.001).

Because the regulation and reorganization of the cytoskeleton, especially MTs and F-actin, greatly affect cell migration [28,29,30], we analyzed the interaction of S100A8/S100A9 with these cytoskeletal components in more detail. First, we focused on a possible interaction of S100A8/S100A9 with F-actin by performing co-sedimentation assays of prepolymerized F-actin in the presence or absence of calcium-free as well as calcium-loaded S100A8/S100A9 (Figure 2A).

The obtained supernatant and pellet fractions showed the binding of S100A8/S100A9 to F-actin in a strictly calcium-dependent manner. In the next series of co-sedimentation assays, we included prepolymerized MTs to study a possible simultaneous interaction of S100A8/S100A9 with both types of filaments as well as the influence of the aforementioned p38-mediated S100A9 phosphorylation [16] (Figure 2B). To be able to detect S100A8/S100A9-mediated cross-linking, the centrifugation conditions were carefully adjusted to sediment MTs while keeping the majority of the lighter, unbound actin filaments in the supernatant fraction. Under calcium-free conditions, no S100A8/S100A9 and only minor amounts of actin were found in the pellet fraction. The intensity of the actin band of this pellet fraction was comparable to that of the MT-free control. In the presence of calcium, S100A8/S100A9 was always clearly detectable in the pellet, regardless of S100A9 phosphorylation. However, the addition of non-phosphorylated S100A8/S100A9 markedly increased the amount of actin in the pellet fraction, whereas the addition of the phosphorylated complex had no effect.

We also conducted fluorescence in vitro assays using fluorescently labeled MTs and actin filaments to visualize and assess the S100A8/S100A9 cross-linking activity (Figure 2C). Adding non-phosphorylated S100A8/S100A9 and calcium induced accumulations of bundles of co-localized and parallel aligned actin filaments and MTs. This bundling and cross-linking of MTs and F-actin was strictly calcium dependent. When S100A8/phospho-S100A9 was used, bundling and cross-linking of MTs and F-actin was completely abrogated (Figure 2B,C), which indicated a regulatory function of S100A9 phosphorylation. To rule out the possibility of the unspecific cross-linking of actin and MTs mediated by the mere presence of S100A8/S100A9 as an additional protein, we performed supplementary experiments using BSA in the presence of calcium as the negative control protein and α-actinin as the positive control. The addition of BSA had no effect on the MTs or actin filaments, whereas adding α-actinin resulted in the formation of bundled actin filaments but no actin-tubulin cross-linking. This results proves the specificity of our findings (Figure 2C).

### 3.3. S100A8/S100A9 Acted as an Actin Filament and MT Cross-Linking Protein in Living Cells, Particularly near the Cell Periphery

**Figure 3:**
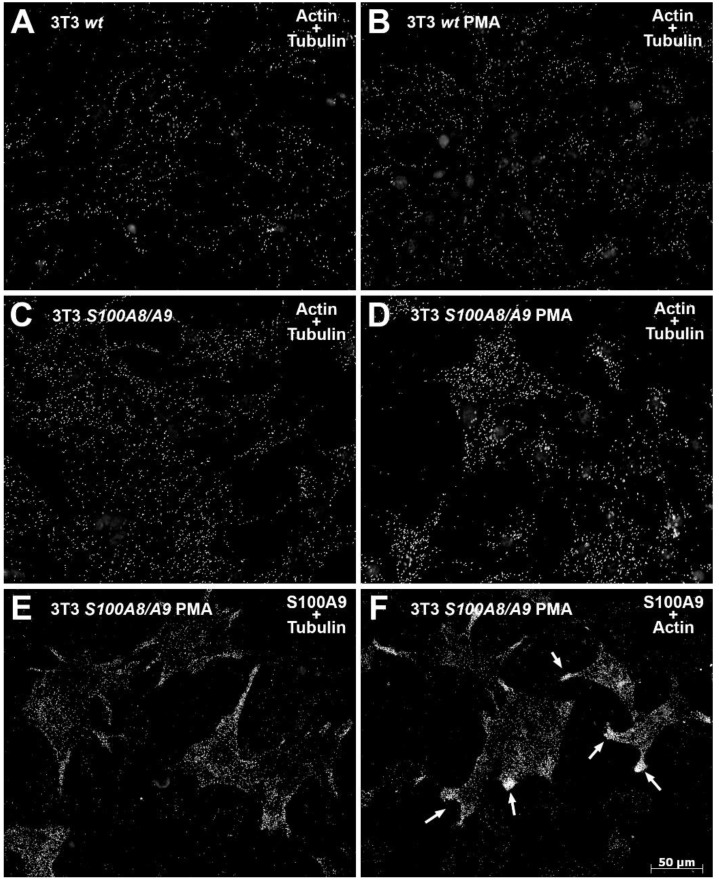
S100A8/S100A9 cross-linked F-actin and MTs in vivo. In the DuoLink^®^ PLA, each spot represents a direct interaction of the two analyzed proteins. In contrast to untransfected fibroblasts (**A**,**B**), the S100A8/S100A9-transfected NIH 3T3 cells (**C**,**D**) showed a higher degree of actin–tubulin interactions. Moreover, the wildtype cells showed no further increase in actin–tubulin interaction upon PMA-stimulation (10 nM/4 h) (**B**), whereas the opposite was found for S100A8/S100A9-expressing NIH 3T3 fibroblasts (**D**). S100A8/S100A9-expressing cells also showed distinct interactions of S100A9 with tubulin (**E**) and F-actin (**F**). Note the accumulation of signal/interaction events in areas of increased membrane dynamics (arrows). Three independent experiments were performed.

In the next set of experiments, we performed DuoLink^®^ PLAs using wildtype and S100A8/S100A9-transfected NIH 3T3 fibroblasts to verify the in vitro findings described above (Figure 3A–F). NIH 3T3 cells were chosen because they are large motile cells with a well-developed cytoskeleton. Generally, we observed more PLA signals of the tubulin-actin antibody combination in the S100A8/S100A9-transfected fibroblasts (Figure 3C) compared to the wildtype cells (Figure 3A), which indicates a higher degree of actin–tubulin interactions. Furthermore, the transgenic cells showed a clear increase in PLA signals upon PMA stimulation (Figure 3D), whereas no obvious changes were observed in the wildtype cells (Figure 3B). The S100A8/S100A9-transfected cells showed distinct and highly localized interactions of the S100A8/S100A9 complex with tubulin (Figure 3E) and actin (Figure 3F) in the PMA-treated cells, as the PLA assays showed clear signal accumulations in areas of increased membrane dynamics and focal adhesion turnover near the cell periphery. The PLA antibody combination mouse anti-S100A8/rabbit anti-S100A9 served as the positive control in S100A8/S100A9-expressing cells and the negative control in the wildtype cells.

### 3.4. Interaction of S100A8/S100A9 with the Cytoskeleton Inhibited Proper Cell Polarization and Migration in Transfected NIH 3T3 Fibroblasts

**Figure 4:**
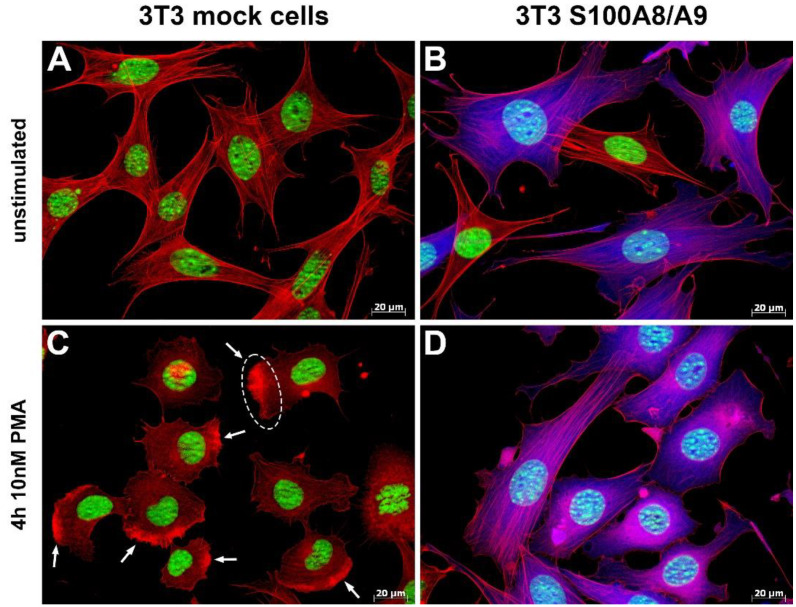
S100A8/S100A9 expression impaired cell polarization and lamellipodia formation. Unstimulated non-expressing control cells (**A**) and S100A8/S100A9-transfected cells (**B**) showed no differences in the overall cell morphology or actin-based cytoskeleton (note: transfection efficiency of S100A8/S100A9-transfected NIH 3T3 cells was not 100%). Upon PMA stimulation (10 nM/4 h), control cells (**C**) exhibited highly increased membrane dynamics (arrows and encircled example area: formation of lamellipodia/membrane ruffles) and spontaneous migration. In contrast, S100A8/S100A9-expressing cells (**D**) failed to polarize properly and to induce lamellipodia formation. Three independent experiments were performed and 25 cells per experiment were analyzed for each individual condition. (red: F-actin, FITC-phalloidin; green: DNA, 4′,6-diamidino-2-phenylindole; blue: anti-S100A9, DyLight649).

Next, we performed stimulation experiments to assess the impact of the observed actin–tubulin cross-linking on the overall cell dynamics (Figure 4A–D).

Again, wildtype and S100A8/S100A9-transfected NIH 3T3 fibroblasts were used. Cells were either stimulated for 4 h with 10 nM PMA or left untreated before being subjected to immunofluorescence microscopy analysis to visualize changes in the actin-based cytoskeleton and morphology. Unstimulated cells of both groups showed no obvious differences and exhibited the characteristic fibroblast morphology and a well-established F-actin cytoskeleton with a high amount of regular stress fibers (Figure 4A,B). Upon PMA stimulation, however, only the wildtype cells showed the expected re-organization of the cytoskeleton, namely, the breakdown of stress fibers, the formation of lamellipodia with a clear leading edge in the direction of migration, and an overall polarization of the cell necessary for migration (Figure 4C). In contrast, S100A8/S100A9-expressing cells failed to polarize and to induce a comparable membrane dynamic. Instead, their overall cell shape remained largely unchanged (Figure 4D).

### 3.5. S100A8/S100A9 Expression Lowered Cell Adhesion by Altering β1-Integrin Clustering and Activation

**Figure 5:**
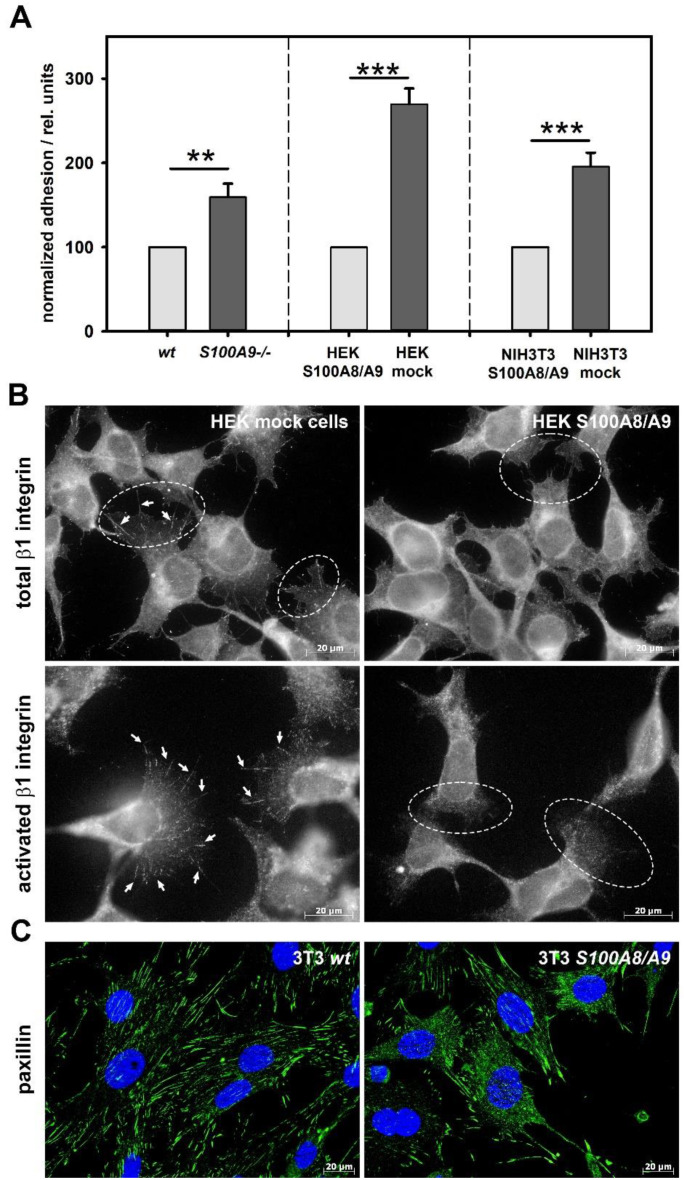
S100A8/S100A9 expression decreased cell adhesion and altered β1-integrin signaling. (**A**) Influence of S100A8/S100A9 expression on cell adhesion in three different cell models. In all studied unstimulated cell populations (murine BMCs isolated from wildtype (wt) and S100A9 -/- mice; S100A8/S100A9-transfected HEK293 or NIH 3T3 cells), the expression of S100A8/S100A9 led to a statistically significant decrease in cell adhesion (*n* = 4; mean ± SD; *** *p* < 0.001; ** *p* < 0.05). (**B**) Immunofluorescence analysis revealed that unstimulated S100A8/S100A9-expressing HEK293 cells showed markedly reduced β1-integrin clustering and β1-integrin activation in comparison to the mock-transfected cells (arrows and encircled example area). Additionally, the expression of S100A8/S100A9 in the NIH 3T3 fibroblasts altered the distribution of the β1-integrin downstream target paxillin (**C**). S100A8/S100A9-transfected NIH 3T3 cells featured fewer and shortened paxillin-rich focal adhesions in comparison to the mock-transfected controls. Instead, paxillin was predominantly distributed throughout the cytoplasm. Three independent experiments were performed and 25 cells per experiment were analyzed for each individual condition.

Because the adhesion of cells to their surrounding substrate is a prerequisite for directed cell migration [23,31], we analyzed the impact of S100A8/S100A9 expression on adhesion in three different cell models: murine BMCs isolated from wildtype and S100A9-deficient mice as primary cells and 100A8/S100A9-transfected HEK293 and NIH 3T3 cells in combination with the corresponding mock-transfected control cells. In all cases, the S100A8/S100A9-expressing cells showed significantly lower adhesion to uncoated plates in comparison to the related non-expressing ones (Figure 5A).

Because adhesion is mostly mediated by integrins, we analyzed the expression and activation of β1-integrins via immunofluorescence microscopy. These studies were performed solely with human HEK293 cells because antibodies specific to activated β1-integrins are only available for the human system. Markedly reduced clustering as well as the activation of β1-integrins was observed in the S100A8/S100A9-expressing HEK293 cells (Figure 5B). Only the control cells showed the characteristic elongated active integrin clusters, which represent stable and mature focal adhesions essential for firm substrate adhesion. Next, we analyzed the intracellular distribution of the integrin signaling downstream target paxillin, which also plays a pivotal role in the formation and regulation of focal adhesions [32,33]. S100A8/S100A9-expressing NIH 3T3 fibroblasts exhibited a mostly cytoplasmic paxillin distribution and, as a consequence, showed fewer and shortened paxillin-rich focal adhesions (Figure 5C), indicating that integrin signaling was affected by S100A8/S100A9 expression.

### 3.6. β1-Integrin CD49d Was Differentially Regulated in S100A8/S100A9-Expressing Cells and Decisively Involved in Mediating Substrate Adhesion

**Figure 6:**
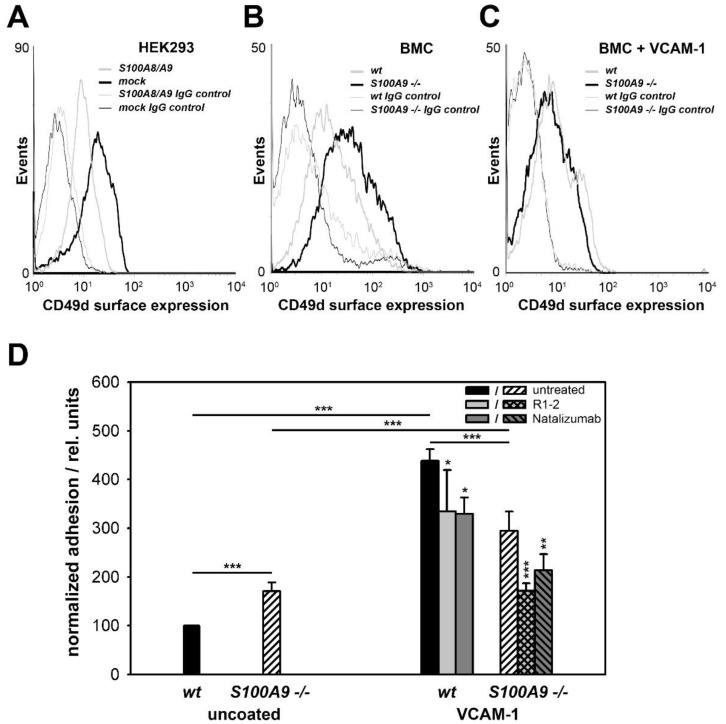
CD49d surface expression was differentially regulated in the S100A8/S100A9-expressing cells, causing a change in substrate adhesion. (**A**–**C**) CD49d surface expression of S100A8/S100A9-expressing HEK293 cells or the murine wildtype (wt) BMCs in the absence (**A**,**B**) or presence (**C**) of the CD49d ligand VCAM-1 compared to the mock transfected HEK293 or S100A9 -/- cells and isotypic controls analyzed by flow cytometry. (**D**) Adhesion assay of murine wildtype and S100A9 -/- BMCs in the absence or presence of VCAM-1 (coating conditions: 1 µg/mL, 16 h, 37 °C). Cells were allowed to adhere for 1 h. In the absence of VCAM-1, S100A9-deficient BMCs showed higher adhesion properties compared to the wildtype cells. However, the wildtype BMCs showed a stronger increase in adhesion in the presence of VCAM-1, which significantly surpassed that of S100A9-deficient cells. Adhesion of both wt as well as S100A9 -/- cells was lowered via CD49d blocking. BMCs from the wildtype and S100A9 -/- mice were preincubated for 30 min with 10 µg/mL of the CD49d blocking antibodies R1-2 or with 10 µg/mL natalizumab, followed by the adhesion assays as described above. All data were normalized to untreated wildtype cells in the absence of VCAM-1 set to 100; *n* = 4; mean ± SD; *** *p* < 0.001; ** *p* < 0.01; * *p* < 0.05; significance levels (asterisks) refer to the comparison to corresponding untreated cells if not otherwise indicated.

To further characterize the observed differences in β1-integrin clustering and activation and to identify significantly involved integrin(s), the surface expression levels of various β1- and β2-integrins or selectins (CD11a (LFA-1), CD11b (Mac1), CD11c (aX), CD18 (β2), CD29 (β1), CD49d (VLA-4α), CD54 (ICAM-1), and CD106 (VCAM-1)) of BMCs and HEK293 cells were screened by flow cytometry. Of all the analyzed adhesion proteins, CD49d was the only one identified to be differentially expressed due to S100A8/S100A9 expression. CD49d represents the alpha subunit of the α4β1 lymphocyte homing receptor VLA-4, which plays an important role in the firm adhesion of neutrophils to the endothelium prior to extravasation [34,35]. Both S100A8/S100A9-transfected HEK293 cells (Figure 6A) and freshly isolated wildtype BMCs (Figure 6B) showed decreased CD49d surface expression levels compared to the corresponding control HEK293 cells or S100A9-deficient BMCs, respectively.

In the presence of the CD49d ligand VCAM-1, the CD49d surface expression was downregulated in S100A9-deficient BMCs, whereas a slight upregulation was observed for the wildtype cells (Figure 6C). To further investigate the involvement and relevance of CD49d in adhesion, we performed blocking studies using the CD49d-specific blocking antibodies R1-2 and natalizumab (Figure 6D). In the presence of VCAM-1, both genotypes showed a strong increase in overall adhesion, with the wildtype adhesion significantly surpassing that of the S100A9-deficient cells with a nearly 5-fold increase compared to about a 2-fold increase in adhesion for the latter. Furthermore, the adhesion of both genotypes was markedly lowered under this condition by the addition of R1-2 or natalizumab.

### 3.7. The Integrin Signaling Downstream Kinases Src and Syk Were Differentially Expressed in Wildtype and S100A9 -/- BMCs

**Figure 7:**
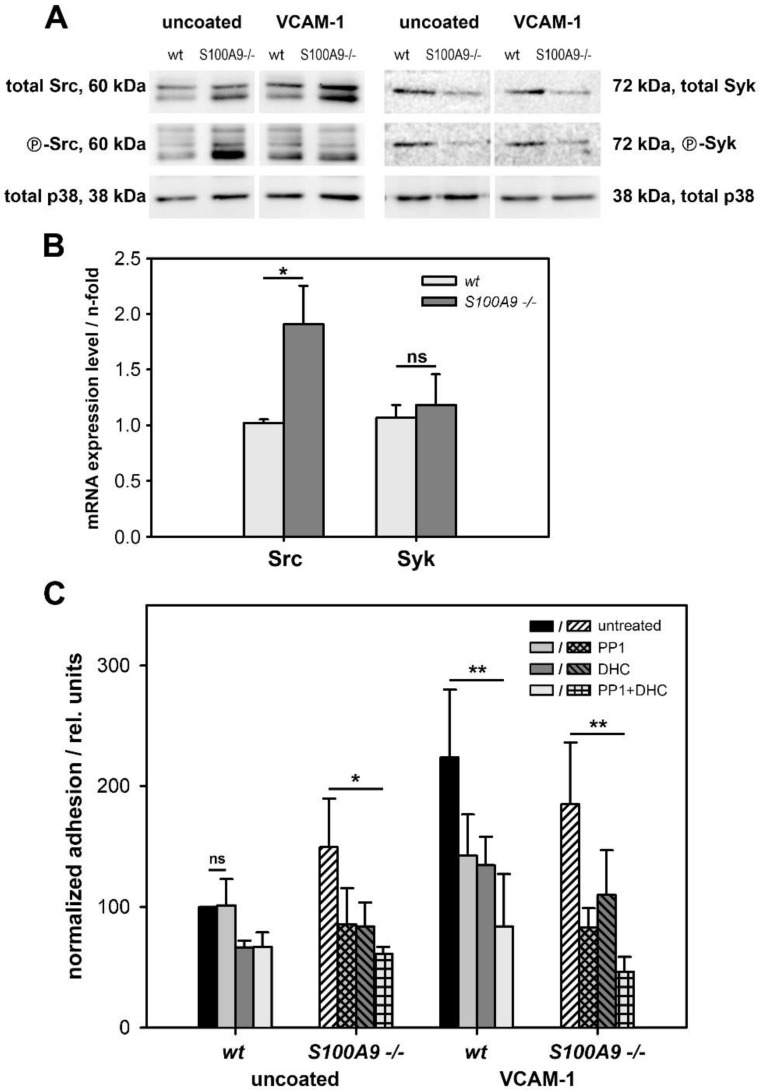
Differential expression and regulation of tyrosine kinases Src and Syk in S100A9 -/- BMCs affected cell adhesion. (**A**) Western blot analysis of total Src and Syk expression as well as Src and Syk activation in the presence or absence of the integrin ligand VCAM-1 after 1 h of adhesion. S100A9 -/- BMCs showed a significantly higher amount of total Src in comparison to the wildtype (wt) cells, while the opposite was found for the Syk expression levels. In the absence of a specific integrin ligand, the wildtype cells showed less Src-416 phosphorylation compared to the S100A9-deficient cells, but in the presence of VCAM-1, it matched the phosphorylation level of S100A9 -/- BMCs. Phosphorylation of Syk was higher in the wildtype cells and independent of VCAM-1. (**B**) RT-PCR analyses of Src and Syk mRNA content confirmed the observed difference in total Src expression, but Syk was found to be equally expressed at the mRNA level. Data were normalized to GAPDH as the housekeeping gene, *n* = 4; mean ± SD; * *p* < 0.05, ns = not significant. (**C**) Adhesion assay (1 h) of the wildtype and S100A9 -/- BMCs in the presence (30 min preincubation) or absence of the specific Src-inhibitor PP1 (10 µM) and/or the specific Syk-inhibitor DHC (2 µM). In the absence of VCAM-1, the addition of either inhibitor led to a decrease in adhesion in S100A9 -/- cells, but the wildtype cells were only affected by Syk blocking. In the presence of VCAM-1, however, both genotypes were equally affected by the addition of the inhibitors and showed a significant decrease in cell adhesion. Data were normalized to untreated wildtype cells in the absence of VCAM-1 set to 100; *n* = 4; mean ± SD; ** *p* < 0.01; * *p* < 0.05, ns = not significant).

Tyrosine kinases of the Src/Syk family play pivotal roles in transmitting integrin-mediated signals via interaction with multiple target proteins, thereby significantly regulating leukocyte arrest, post-arrest strengthening, and transmigration [35,36,37,38,39]. Therefore, we examined the grade of activation of these two kinases in relation to their total expression level via Western blot analyses (Figure 7A).

The experiments were performed using freshly isolated wildtype and S100A9 -/- murine BMCs, which were allowed to adhere for 1 h in the presence or absence of VCAM-1. Labeling of total p38 served as the control to ensure equal protein loading. We observed pronounced differences in the total expression level of Src and Syk between the two genotypes, with S100A9 -/- BMCs exhibiting much higher Src expression compared to that of the wildtype cells. In contrast, Syk expression was higher in the wildtype cells. Differences in Src expression were confirmed by RT-PCR analysis. No significant difference in mRNA level was found for Syk expression, indicating that post-transcriptional regulation occurred (Figure 7B). Although the observed differences in total Src/Syk content make it difficult to evaluate and directly compare the ratios of the activated/phosphorylated kinases, overall, the S100A9 -/- BMCs showed a higher amount of activated Src. In the presence of VCAM-1, however, Src-phosphorylation was only increased in the wildtype cells, in which it matched that of the S100A9-deficient cells. In contrast, Syk-phosphorylation was always higher in the wildtype BMCs, independent of the presence of VCAM-1.

To further investigate the possible consequence of the observed alterations in Src and Syk regulation as well as the relevance of both kinases in the adhesion process, we performed blocking studies using the Src-specific inhibitor PP1 and the Syk-specific inhibitor DHC (Figure 7C). In the absence of VCAM-1, the addition of PP1 only affected the S100A9 -/- cells, causing a significant decrease in adhesion. In contrast, both genotypes showed decreased adhesion after the inhibition of Syk or the simultaneous blocking of Syk and Src. In the presence of VCAM-1, however, both wildtype and knockout cells were equally affected by the addition of the inhibitors and showed a significant decrease in cell adhesion to nearly comparable levels.

### 3.8. S100A8/S100A9 Expression Affected Migratory Properties of Cells

**Figure 8:**
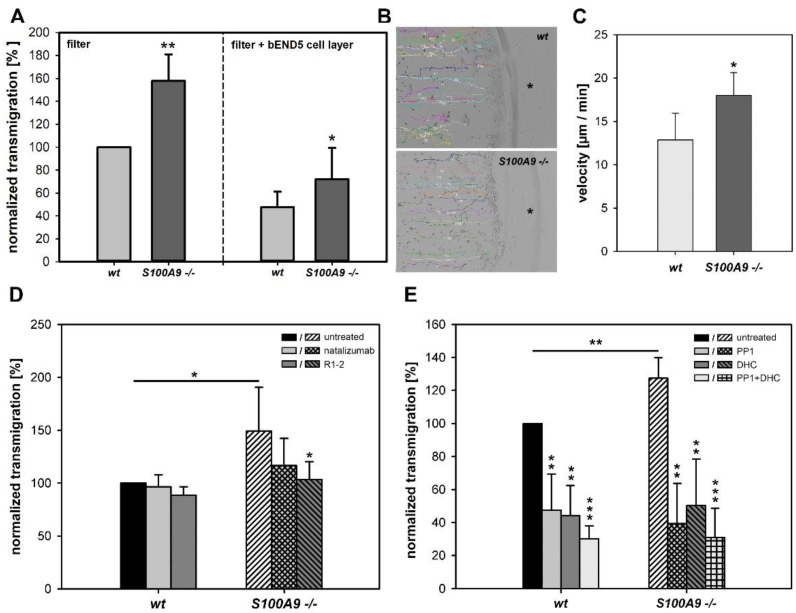
S100A8/S100A9 expression altered transmigration rates. Two chamber filter-based transmigration assays of the wildtype (wt) and S100A9 -/- BMCs in the absence (**A** left and **D**,**E**) or presence (**A** right) of a bEND5 endothelial cell layer (*n* = 5). S100A8/S100A9-deficient cells showed increased numbers of transmigrated cells after 4 h of migration. (**B**) Migration tracks of isolated BMCs analyzed from the 2D migration assays showed no differences in cell orientation behavior between the wildtype and S100A9-deficient cells during chemotaxis toward the source of the chemoattractant (asterisk) (*n* = 3). (**C**) S100A9-deficient BMCs showed a significantly higher overall migration speed compared to the wildtype cells (*n* = 3; total number of tracked cells = 69 wildtype and 57 S100A9 -/-). (**D**) The addition of natalizumab (10 µg/mL) or R1-2 (10 µg/mL) reduced the number of transmigrated S100A9-deficient cells to the wildtype levels, whereas the wildtype cells were not affected (*n* = 4). (**E**) Dependency of the transmigration rates on Src (10 µM) and Syk (2 µM) activities. Addition of either inhibitor (PP1/DHC) or the combination of both resulted in a significant decrease in transmigrated cells and reduced the otherwise higher transmigration of S100A9-deficient cells to the wildtype levels (*n* = 3, mean ± SD; *** *p* < 0.001; ** *p* < 0.01; * *p* < 0.05. (**D**,**E**) All data were normalized to the untreated wildtype cells set to 100%, and the significance levels (asterisks) refer to the comparison to the corresponding untreated cells if not otherwise indicated.

Because cell adhesion has a strong impact on directed cell migration [23,31], we analyzed the effect of S100A8/S100A9 expression on migration by performing filter-based transmigration (Transwell) and 2D migration assays using freshly isolated murine wildtype and S100A9 -/- BMCs. First, we conducted filter-based transmigration experiments in the presence or absence of a bEND5 endothelial cell layer as an additional barrier to assess the rate and ability of spontaneous cell transmigration (Figure 8A).

In both cases, the wildtype cells showed fewer transmigrated cells in comparison to the S100A9 -/- cells. To check whether this difference was caused by an alteration in cell chemotaxis during migration, we performed 2D under-agarose migration studies, monitored the migration paths of the cells toward the source of the chemoattractant (Figure 8B), and determined the average migration speed of the cells (Figure 8C). Analyses of the migration paths showed no differences in chemotactic behavior between the two genotypes. Both wildtype and S100A9 -/- cells moved straight and continuously toward the source of the chemoattractant (Figure 8B). Analysis of the average migration velocity, however, revealed that S100A9 -/- BMCs were able to move significantly faster than the wildtype cells (Figure 8C). In the next step, we investigated the importance and involvement of CD49d in the transmigration process. We conducted filter-based transmigration assays with and without the addition of the CD49d-specific inhibitors R1-2 or natalizumab (Figure 8D). The addition of natalizumab or R1-2 reduced the number of transmigrated S100A9-deficient cells to the wildtype levels, whereas the wildtype cells were not adversely affected.

In the last set of experiments, we investigated the involvement of the tyrosine kinases Src and Syk in the transmigration process via filter-based transmigration assays with and without the addition of the Src-inhibitor PP1 or the Syk-inhibitor DHC (Figure 8E). The addition of either inhibitor alone as well as their combined application resulted in a significantly lower amount of transmigrated cells for both genotypes and reduced the otherwise higher transmigration rate of the S100A9 knockout cells to wildtype levels.

## 4. Discussion

The family of S100 proteins is the largest subgroup of calcium-binding proteins, and S100A8 and S100A9 are the major calcium-binding proteins of neutrophils and monocytes [11]. Most previous S100A8/S100A9 studies were focused on elucidating the extracellular functions of these proteins [13,14,40,41,42,43], and much less is known about the intracellular role of this protein complex. One already described important function of S100A8/S100A9 is its direct interaction with MT to promote polymerization and bundling, but to date, this has only been shown in cell-free in vitro experiments using purified S100A8/S100A9 complexes and tubulin [16].

Therefore, we established stable-transfected S100A8/S100A9-expressing HEK293 and NIH 3T3 cell lines to analyze the intracellular function of S100A8/S100A9 in more detail in a living cell system. Using HEK293 cells, we were able to confirm previous results reported for tubulin interaction with S100A8/S100A9 in human monocytes [19]. Our data also indicate that S100A8/S100A9 not only promotes tubulin polymerization, but it also stabilizes MTs in vivo, thus affecting the overall MT dynamics. This result is of biological relevance, as both of these characteristics (MT polymerization and stability) have a significant impact on the overall dynamic remodeling of the cytoskeleton [44,45], which in turn is a prerequisite for locomotion [30,45,46]. Furthermore, because the MT cytoskeleton of the resting S100A8/S100A9-expressing and non-expressing cells did not differ, our data suggest that S100A8/S100A9 predominantly affects dynamic MTs during phases of growth or shrinkage as opposed to “stationary” ones, and is thus not primarily involved in maintaining the steady-state of the MT cytoskeleton. This observation points to a function of S100A8/S100A9 in the coordination and modulation of the activation of cell dynamics. The stimulation of HEK293 cells or primary bone marrow cells by the phorbol ester PMA caused the cross-linking of microtubules and actin filaments via S100A8/S100A9 complexes. However, PMA is also well-known as an activator of protein kinase C, which in turn leads to the phosphorylation of integrins, resulting in changes in the integrin conformation and interaction with the cytoskeleton and downstream adaptor molecules. Therefore integrin phosphorylation provides a trigger and initiates rapid cell adhesion and signaling events [47].

Because cell migration is mainly dependent on F-actin dynamics, we analyzed interactions of S100A8/S100A9 with MTs and actin. Co-sedimentation assays as well as in vitro and in vivo experiments revealed not only an interaction of S100A8/S100A9 complexes with F-actin, as reported earlier [20], and with MTs [16], but also a novel function of S100A8/S100A9 as a calcium-dependent MT/F-actin cross-linker that efficiently bridges both types of filaments. Additionally, our data show that the binding of S100A8/S100A9 to MTs and to F-actin is strictly calcium-dependent, whereas the phosphorylation of S100A9 alters the affinity toward F-actin, as suggested by Lominadze et al. (2005) [20], and more importantly, modulates the cross-linking activity of the S100A8/S100A9 complex. This cross-linking activity was completely abrogated by phosphorylated S100A8/S100A9 complexes, which is analogous to previous findings about MT bundling [16].

MTs also play an important role in the targeted delivery and transportation of numerous proteins and in the coordination of extending the lamellipodia of moving cells [48,49,50,51]. They are also involved in the formation and turnover of focal adhesions, as they are known to “contact” these structures, thereby leading to their disassembly [52,53]. Consequentially, we hypothesize that the cross-linking activity of S100A8/S100A9 described herein directly influences the targeted delivery and distribution of proteins that are critically involved in cell polarity or the composition of focal adhesions such as integrins, thereby also affecting overall integrin activation, distribution/clustering, and subsequent signaling. Thus, S100A8/S100A9 may promote the breakdown and recycling of these structures and prevent the maturation of nascent focal complexes to focal adhesions. This premise is supported by the results of the DuoLink^®^ PLA assays, which revealed the presence of S100A8/S100A9 in areas where membrane protrusions/lamellipodia were formed.

In summary, these data point to a local submembraneous mechanism that could help phagocytes maintain a non-adherent resting state until secondary signaling cascades activate kinases such as p38 MAPK, for which an interaction with S100A9 was already reported [16,20], and abrogate the cross-linking by phosphorylating S100A9 on threonine113. In the S100A8/S100A9-deficient BMCs, this accurate control of overall cell activation was markedly impaired, leading to increased cell dynamics, as indicated by disordered cell adhesion and uncontrolled migration rates. S100A8/S100A9 may also act as a formin-related protein that facilitates the guidance of MTs along actin filaments toward focal adhesions and supports their stabilization by linking them to the actin-rich substructure of focal adhesions or focal complexes [54,55,56].

MTs have been shown to contact focal adhesion points, leading to their disassembly. It is therefore feasible to assume that the interplay of S100A8/S100A9 with the MT cytoskeleton has a measurable influence on cell adhesion, and the regulation and modulation of adhesion, in turn, affect directed cell migration. In fact, S100A8/S100A9-expressing cells showed a significant reduction in substrate adhesion that was caused by alterations in the surface expression and the activation of β1-integrins. They were further characterized by a redistribution of the integrin signaling downstream target paxillin and by altered regulation of the target kinases Src and Syk. In particular, we identified CD49d as being differentially regulated in S100A8/S100A9-expressing BMCs.

CD49d is the alpha subunit of the α4β1 lymphocyte homing receptor VLA-4, and it plays an important role in the adhesion of leukocytes to the endothelium prior to extravasation [34,57]. It supports lymphocyte rolling in vivo in venules of the central nervous system. Although CD49d is often described as playing only a minor role in neutrophil adhesion compared to CD11b/CD18 (Mac1), our data indicate that S100A8/S100A9 keeps the cells in a resting state via the modulation of CD49d signaling to prevent uncontrolled extravasation. However, we found that the regulation of CD49d surface expression was dependent on both S100A8/S100A9 expression and on the presence of the extracellular ligand VCAM-1 [35]. This finding suggests that S100A8/S100A9 has a strong impact on the modulation of target-oriented integrin activation and signaling, whereas the loss of S100A8/S100A9 leads to impaired regulation and inappropriate cell activation, which finally results in the overall higher dynamic activity of S100A9 -/- phagocytes.

The proposed dysregulation of integrin signaling is supported by our finding that both expression and activation of the downstream target kinases Src and Syk were affected by the loss of S100A8/S100A9. Functional consequences of the observed alterations in Src/Syk expression and activity were in line with the results of subsequent blocking studies. We found that S100A9 -/- cells were prone to Src and Syk blocking, both in the presence and absence of VCAM-1, whereas the wildtype cells were only affected in the presence of the integrin ligand. The observed alterations in integrin-mediated adhesion had a direct impact on cell migration, as demonstrated by filter-based transmigration and 2D migration assays. This is a biologically relevant consequence that is important for the in vivo function of phagocytes to quickly reach sites of inflammation upon stimulation while otherwise remaining dormant. S100A9-/- BMCs exhibited an overall higher rate of spontaneous transmigration, whereas chemotactic orientation of both genotypes was comparable.

We also illustrated the significant influence of integrin (particularly CD49d) signaling on spontaneous transmigration via blocking studies using natalizumab or R1-2. Our new data support recent findings of a new extracellular function of S100A8/S100A9 on murine monocytes, whereby missing extracellular S100A8/S100A9 causes the preactivation of these cells. This effect is not linked to proinflammatory S100A8/S100A9 dimers, and is exclusively restricted to calcium-induced tetramers of S100A8/S100A9 binding and activating CD69 on monocytes [58]. We also recently showed that the contact of neutrophils with the endothelium leads to a rapid S100A8/S100A9-dimer release, and that these dimers in turn bind in an autocrine manner to the neutrophil’s Toll-like receptor 4 and initiate the activation of β2-integrins from a low-affinity to an intermediate-affinity state. This process leads to the firm arrest of the cells and facilitates their extravasation [42].

Clearly, intracellular and extracellular S100A8/S100A9 complexes have synergistic regulatory functions that prevent unwanted and/or uncontrolled phagocyte activation. Accordingly, the inhibition of Src and Syk activity was able to reduce the otherwise higher transmigration rate of knockout BMCs to the wildtype level. The function of integrins critically depends on their conformational state. During rolling along the endothelium, integrins undergo conformational changes from a low-affinity bent conformation to an intermediate, and finally to a high-affinity open conformation, which eventually allows for firm adhesion of the phagocytes and their extravasation without any changes in the overall expression levels of these molecules [59]. In our study, we only detected differences in the CD49d expression levels between the S100A8/S100A9-expressing cells compared to the non-S100A8/S100A9-expressing cells. We currently cannot rule out the possibility that the functions of other β1- or β2-integrin molecules might also be affected by missing S100A8/S100A9, despite the lack of changes in their overall expression levels.

In summary, the results presented herein provide novel insights into the molecular mechanisms necessary to precisely control the regulation of phagocyte activation, adhesion, and migration. We showed that intracellular S100A8/S100A9, which was already known to integrate two major activation pathways (MAPK and calcium-dependent signaling), plays a pivotal role in cytoskeletal dynamics. Moreover, we demonstrated for the first time that S100A8/S100A9 cross-links MTs and actin filaments in a calcium- and phosphorylation-dependent manner. As a consequence, the modulation of cytoskeletal dynamics by S100A8/S100A9 affects the overall cell adhesion, proper cell polarization, and, as a result, transmigration. In contrast, the absence of intracellular S100A8/S100A9 leads to dysregulated and increased cell activation. Thus, S100A8/S100A9 promotes the maintenance of a resting/quiescent phagocyte state. The observed cross-linking activity of S100A8/S100A9 is strictly phosphorylation-dependent, so pharmacologic interference with this process may be an interesting and promising approach to restrict phagocyte activation in inflammatory diseases.

## Data Availability

All data associated with this study are available in the main text or are available through the corresponding author upon request.
